# The *S. pombe* CDK5 Orthologue Pef1 Cooperates with Three Cyclins, Clg1, Pas1 and Psl1, to Promote Pre-Meiotic DNA Replication

**DOI:** 10.3390/biom11010089

**Published:** 2021-01-12

**Authors:** Shinya Matsuda, Ushio Kikkawa, Akio Nakashima

**Affiliations:** 1 Biosignal Research Center, Kobe University, 1-1, Rokkodai-cho, Nada-ku, Kobe 657-8501, Japan; ukikkawa@kobe-u.ac.jp; 2 Department of Pharmaceutical Sciences, University of Shizuoka, 52-1 Yada, Suruga-ku, Shizuoka 422-8526, Japan; 3 Department of Bioresource Science, Graduate School of Agricultural Science, Kobe University, Kobe, 1-1, Rokkodai-cho, Nada-ku, Kobe 657-8501, Japan

**Keywords:** meiosis, Pef1, CDK5, Cdc2, pre-meiotic DNA replication, cyclin

## Abstract

Meiosis is a specialized cell division process that mediates genetic information transfer to the next generation. Meiotic chromosomal segregation occurs when DNA replication is completed during the pre-meiotic S phase. Here, we show that *Schizosaccharomyces pombe* Pef1, an orthologue of mammalian cyclin-dependent kinase 5 (CDK5), is required to promote pre-meiotic DNA replication. We examined the efficiency of meiotic initiation using *pat1-114* mutants and found that, meiotic nuclear divisions did not occur in the *pef1Δ pat1-114* strain. Deletion of *pef1* also suppressed the expression of DNA replication factors and the phosphorylation of Cdc2 Tyr-15. The double deletion of *clg1* and *psl1* arrested meiotic initiation in *pat1-114* mutant cells, similar to that of *pef1*-deficient cells. Meiotic progression was also slightly delayed in the *pas1*-deficient strain. Our results reveal that Pef1 regulates cyclin-coordinated meiotic progression.

## 1. Introduction

Sexual reproduction is an important process that mediates genetic information transfer to the next generation in many species, including yeast and mammals. Gametes form through meiotic cell division, which is accomplished by DNA synthesis, during the pre-meiotic S phase followed by two consecutive rounds of nuclear division (meiosis I and meiosis II). Meiosis results in gene diversity in gametes primarily through homologous recombination at specific genome regions known as ‘hot spots.’ Homologous recombination takes place due to repair of DNA damage caused by self-inflicted double-strand breaks (DSBs) [1]. In response to the accumulated unrepaired DSBs, checkpoint machinery arrests or delays the meiotic cell cycle, preventing misregulation of chromosomal segregation. Thus, precise DNA replication and DSB repair are essential for initiating meiotic nuclear division [2]. 

The fission yeast *Schizosaccharomyces pombe* is an excellent model organism for meiosis research because its process of meiotic cell division is similar to that of mammalian cells. Nitrogen depletion leads to the accumulation of Mei2, a master regulator of meiotic initiation, resulting in the arrest of mitosis and initiation of the meiotic cell cycle [3,4,5]. As one of the well-known regulatory systems of meiosis in *S. pombe*, the Pat1—Mei2 pathway is considered to be an important checkpoint for the transition between mitosis and meiosis. In fact, Pat1 kinase mediates blocking the Mei2 phosphorylation, compulsively initiating meiosis [4] and meiosis is easily induced by inactivating Pat1 kinase. This characteristic of *S. pombe* allows us to research molecular mechanisms of meiosis [6].

Cyclin-dependent kinase (CDK) is a major cell cycle regulator whose activity is regulated by its activating subunits, known as cyclins [7]. During meiosis in *S. pombe*, Cdc2 (equivalent to mammalian CDK1) helps regulate various aspects of meiosis, including meiotic DNA replication, DSB formation and recombination [8,9]. Thus, alterations in the Cdc2 activity level significantly influence meiotic cell cycle efficiency. Although the deletion of G1 cyclins (Cig2, Cig1 and Puc1) and meiotic-specific cyclins (Rem1 and Crs1) deprives cells of the ability to initiate meiosis, meiosis is efficiently driven by the introduction of high copy Cdc2-Cdc13/cyclin B fusion protein into these cyclin-deficient cells [10]. Therefore, elucidating the regulatory mechanisms of Cdc2 activity is important in understanding meiosis.

Other kinases or phosphatases also modulate CDK activities. As a widely accepted regulatory model in mitosis, Cdc2 activity is inhibited by the Wee1 kinase-mediated phosphorylation of Tyr-15, whereas Cdc25 phosphatase increases Cdc2 activity by dephosphorylating Tyr-15 and consequently promotes G2/M transition in mitosis [7,8]. In meiosis, Cdc2 Tyr-15 phosphorylation increases during the pre-meiotic S phase until DNA replication is completed and its dephosphorylation allows cells to enter the meiosis I phase [8]. Similar to *S. pombe*, mouse CDK1 inactivation by Wee1B-dependent phosphorylation arrests meiosis in oocytes and arrested meiosis is resumed by Cdc25B-driven de-phosphorylation of Tyr-15 [11,12]. Moreover, in *Xenopus* oocytes, Wee1 downregulation turns off excess DNA synthesis, thereby ensuring continuous meiotic cell division without the S phase intervention [13]. Therefore, the phosphorylation state of Cdc2 is an important marker of meiosis I entry in many species. However, the regulatory mechanism of Cdc2 phosphorylation during meiosis has not yet been fully elucidated.

*S. pombe* Pef1 is an orthologue of mammalian CDK5, a unique kinase that plays diverse roles in terminally differentiated cells such as neurons [14,15]. Although Pef1 interacts with three types of cyclins: Clg1, Pas1 and Psl1 [16,17], the physiological functions and activation mechanisms of Pef1 are poorly understood. We recently reported that Pef1-Clg1 and Pef1-Pas1 complexes regulate the initiation of sexual differentiation by controlling the balance among the TORC1, autophagy and Ste11-Mei2 pathways [18]. We have also shown that Psl1 stably interacts with Pef1 during nitrogen starvation; however, the Pef1-Psl1 complex’s role is still unknown. Regarding the elucidation of Pef1-Psl1 function, we have previously demonstrated that *psl1* and *pef1* are required for the mating process [18]. This finding implies that the Pef1-Psl1 complex regulates nuclear functions such as transcription, chromosomal segregation and DNA replication. However, the role of Pef1 and these cyclins in meiosis remains unclear. 

In this study, we investigated whether Pef1 is involved in the regulation of the meiotic cell cycle. Using *pat1-114* temperature-sensitive (ts) mutant strains, which can induce meiosis by shifting the permissive temperature (25 °C) to a restrictive temperature (34 °C), we reveal that Pef1 is required for Cdc2 Tyr-15 phosphorylation and DNA replication during the pre-meiotic S phase. Furthermore, our data provide evidence that Clg1, Psl1 and Pas1 are also required for meiotic progression. Thus, we propose that Pef1–cyclin complexes regulate meiotic progression.

## 2. Materials and Methods 

### 2.1. Yeast Strains, Growth Conditions and Induction of Meiosis

The *S. pombe* strains used in this study are listed in Table 1. The cells were cultured, as previously described [18]. In brief, the cells were precultured in yeast extract with supplements (YES) medium or Edinburgh’s minimal medium (EMM), which contained 20 mg/mL glucose as a carbon source and 500 µg/mL ammonium chloride as a nitrogen source, at 25 °C overnight under shaking at 110–120 rpm. To synchronize the cells in the G1 phase, a series of *pat1-114* ts mutants were grown in EMM at 25 °C under shaking at 90 rpm. After they reached an OD_595_ of 0.6–0.8, the cells were transferred to EMM-NH_4_Cl, a nitrogen-free medium and then incubated at 25 °C under shaking at 90 rpm for 14–16 h. G1-synchronized cells were incubated at 34 °C to initiate meiosis. To monitor the cell growth rate, five-times serially diluted cultures were spotted on EMM agar and incubated at 25 °C, 34 °C or 37 °C. General and molecular genetic techniques were conducted following standard protocols [19,20,21]. To generate heterozygous diploids, the haploid strains were grown in YES medium. The cultures were mixed and resuspended in sterile water. The cell mixture was then spotted onto sporulation agar with amino acid supplements (SPAS) plates (10 mg/mL glucose, 1 mg/mL KH_2_PO_4_, vitamins and 45 mg/L adenine, leucine, uracil, histidine and lysine) and the plates were incubated at 25 °C overnight. This process encourages the haploids to mate and form diploids. To isolate the diploids, the mated cell mixture was plated onto YES medium containing hygromycin B and nourseothricin and the plates were incubated at 33 °C.

### 2.2. Construction of Modified Strains and Gene Expression Plasmids

Direct chromosomal integration methods were performed. as previously described [18]. In brief, a 3HA-hphMX cassette was integrated before the terminal codons of the *pef1* open reading frame (ORF). For gene disruption, the kanMX, natR and hphMX cassettes were replaced with the individual ORFs [22,23]. Construction of the expression plasmids, *pef1^+^*, *pef1^T13A^*, *pef1^Y14F^* and *pef1^K32R^*, was performed as previously described [18].

### 2.3. Flow Cytometry Analysis

Meiosis-induced cells were fixed with 70% ethanol and then washed with 50 mM sodium citrate twice. The cells were resuspended in 50 mM sodium-containing 0.1 µg/mL RNase and incubated at 37 °C for 2 h. RNase-treated cells were stained with propidium iodide (final concentration: 2 µg/mL) to measure their DNA content using a BD FACS Accuri flow cytometer (BD Bioscience, San Jose, CA, USA). BD Accuri C6 software was employed to measure the DNA contents.

### 2.4. Protein Preparation and Immunoblotting

Protein preparation, immunoprecipitation and immunoblotting were performed as previously described [18]. Cell cultures were mixed with 7% trichloroacetic acid and put on ice for a minimum of 10 min. After centrifugation, the cell pellets were washed twice with cold acetone and dried. The cells were lysed in buffer A [50 mM Tris-HCl (pH 7.5), 150 mM NaCl, 5 mM ethylenediaminetetraacetic acid (EDTA), 10% glycerol, 20 mM β-glycerophosphate, 0.1 mM Na_3_VO_4_, 10 mM p-nitrophenyl phosphate (p-NPP) and 10 mM NaF] containing 0.2% NP-40 and glass beads and then vortexed. Whole-cell extracts were mixed with 3 × SDS sample buffer and boiled for 5 min. The supernatants of these boiled lysates were separated by SDS-PAGE and then subjected to Western blotting. Anti-α-tubulin (B5-1-2) antibody was obtained from Sigma. Mouse anti-HA (12CA5) antibodies were acquired from Roche. Anti-PSTAIRE antibody was purchased from Santa Cruz. Anti-phospho-Cdc2 Tyr-15 antibody was purchased from Cell Signaling Technology. After incubation with HRP-conjugated secondary antibodies, proteins were detected using the ECL Plus Western Blotting Substrate (Thermo Scientific). The immunoblot band intensities were measured by Image J software (NIH).

### 2.5. RNA Preparation and Reverse-Transcription Quantitative Real-Time PCR (RT-qPCR)

RNA preparation and Reverse-transcription Quantitative Real-Time PCR (RT-qPCR) were conducted as previously described [18]. RNA was prepared by disrupting the cells with glass beads in buffer C [0.2 M Tris-HCl (pH 7.5), 0.5 M NaCl, 10 mM EDTA and 1% SDS]. Proteins were removed using a 25:24:1 phenol:chloroform:isoamyl alcohol mix (pH 5.2) (Nakarai Tesque, Kyoto, Japan) and RNA was precipitated with 100% ethanol. For qPCR, 0.5 μg of total RNA was reverse-transcribed to cDNA using ReverTra Ace qPCR RT Master Mix (TOYOBO, Osaka, Japan). qPCR was performed using a LightCycler 480 (Roche Diagnostics, Mannheim, Germany) and a Thunderbird SYBR qPCR Mix Kit (TOYOBO). The primer sequences used are shown in Table 2.

### 2.6. Fluorescence Microscopy

To calculate the percentages of meiotic cells, the cells were treated with 70% ethanol and then stained with DAPI. Pre-meiosis, meiosis I, meiosis II and ascus were distinguished using fluorescence microscopy (BZ-8000, Keyence, Osaka, Japan). The percentages of cells in meiosis I and meiosis II were calculated as a ratio of the number of meiotic cells to total cells.

### 2.7. Statistical Analysis

The data are shown as the mean ±SEM of the indicated number of observations. To determine the significant differences, two-way analysis of variance (ANOVA) was performed using Prism ver. 8. *p* values < 0.05 were considered statistically significant.

## 3. Results

### 3.1. Pef1 is Required for Meiotic Progression

**Figure 1:**
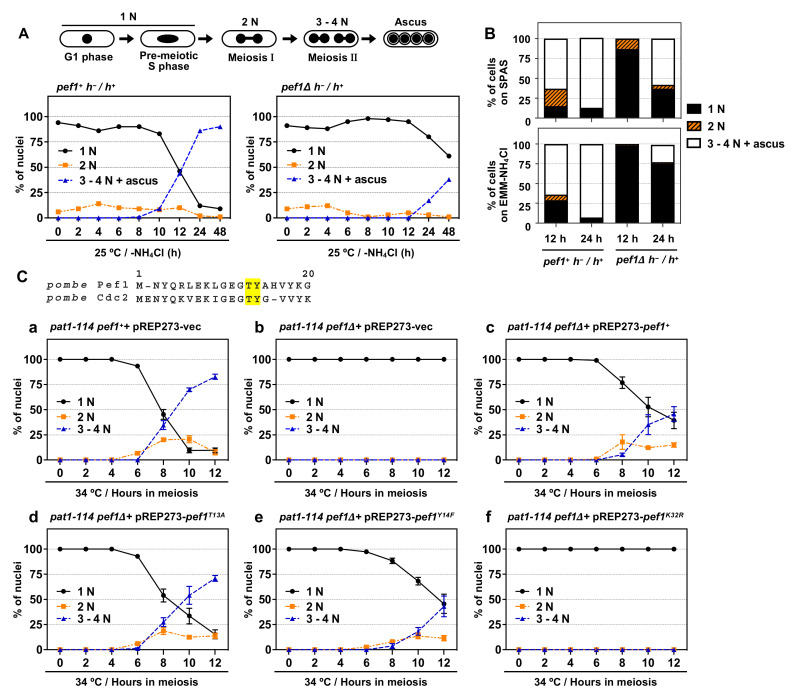
*pef1* is required for meiotic progression. (**A**,**B**) Illustration representing alterations in the number of nuclei during meiosis in *S. pombe*. Diploid *wt* (AN0353) and *pef1Δ* (MS100) cells were cultured in YES at 33 °C and then the cells were transferred to EMM–NH_4_Cl liquid medium or spotted on SPAS or EMM–NH_4_Cl agar medium. After incubating the cells for the indicated times at 25 °C, they were fixed, stained with DAPI and observed using fluorescence microscopy. The graph indicates the percentages of G1-pre-meiotic S phase (1N, black solid line), meiosis I (2N, orange broken line) and meiosis II and ascus (3–4N, blue broken line). At least 100 cells were counted at each time. (**C**) Illustration representing N-terminal amino acid alignment of Pef1 with Cdc2. *pat1-114 pef1^+^* (FY7122) or *pat1-114 pef1Δ* (MS254) haploid cells were transformed with pREP273-empty vector (Ca, Cb), pREP273-*pef1^+^* (Cc), pREP273-*pef1^T13A^* (Cd), pREP273-*pef1^Y14F^* (Ce) or pREP273-*pef1^K32R^* (Cf). Exponentially grown cells were incubated in EMM–NH_4_Cl for 14–16 h at 25 °C. Synchronous meiosis was induced by shifting the cultures from a permissive temperature (25 °C) to a restrictive temperature (34 °C). Then, the cells were fixed at the indicated times, stained with DAPI and observed using fluorescence microscopy. The graph indicates the percentages of cells in the G1-pre-meiotic S phase (1N), meiosis I (2N) or meiosis II (3–4N). At least 100 cells were counted each time. Results are expressed as means ±SEM from three independent experiments.

*S. pombe* Cdc2 is known to play a pivotal role in meiotic initiation and progression [8] but the roles of other CDK family members in meiosis are less well understood. We previously found that *pef1*-deficient homothallic haploid cells have a low mating ability on SPAS (sporulation agar with amino acid supplements) and EMM–NH_4_Cl agar medium (a minimal nutrition medium without nitrogen sources) [18]. Thus, we hypothesize that Pef1 regulates meiotic progression. To verify this hypothesis, we constructed a *pef1Δ* diploid strain. As shown in Figure 1A, wild-type diploid cells (*pef1^+^ h^−^/h^+^*) effectively induced meiosis after 10–12 h of nitrogen starvation and finally formed an ascus 24–48 h later. In contrast, the efficiency of meiotic progression and ascus formation decreased in *pef1*-deficient diploid cells compared with wild-type diploid cells. The efficiency of meiotic induction on SPAS and EMM–NH_4_Cl agar medium also decreased by *pef1* deletion (Figure 1B and Figure S1A). These results support our hypothesis that Pef1 is required for meiotic progression.

The synchronous meiotic induction in the *pat1-114* temperature-sensitive (ts) mutant is one of the most helpful methods to examine the efficiency of meiotic progression; thus, we adopted this system to evaluate the effect of *pef1* deletion on the efficiency of meiotic progression. First, we constructed *pef1Δ pat1-114* ts mutant strains that possessed leucine auxotrophy to introduce pREP273-vectors, including ORFs of the *pef1* wild-type and *pef1* mutants. A *pat1-114 pef1^+^* strain with a pREP273-empty vector (*pat1-114 pef1^+^+* pREP273-vec) was used as a control. We confirmed that these strains’ cells exhibited growth defects at high temperatures (34 °C and 37 °C), indicating that *pef1* deletion does not influence the temperature sensitivity of *pat1-114* (Figure S1B). To induce synchronous meiosis, we synchronized cells at the G1 phase by nitrogen starvation at a permissive temperature (25 °C) and then the incubation temperature was shifted to the restrictive temperature (34 °C) to initiate meiosis. As shown in Figure 1 (Ca) and Figure S1C, the control cells efficiently entered into meiosis I (observed as binuclear cells: 2N) and meiosis II (observed as cells with three or four divided nuclei: 3–4N) after 6–8 h of the temperature shift. The meiotic nuclear division did not occur in *pef1*-deficient *pat1-114* cells (Figure 1(Cb)), indicating that Pef1 is required for the initiation of meiotic nuclear division. Additionally, the expression of Pef1 WT could moderately rescue the meiosis arrest of *pef1*-deficient cells (Figure 1(Cc)), whereas Pef1 K32R, a kinase-dead mutant [18], was unable to restore the arrest (Figure 1(Cf). Interestingly, although the expression of Pef1 T13A, a non-phosphorylatable mutation, efficiently promoted meiotic progression (Figure 1(Cd)), the efficiency of meiotic progression in Pef1 Y14F-expressing cells (Figure 1(Ce)) was slightly delayed compared to Pef1 WT-expressing cells. Taking these results together, we can conclude that the kinase activity of Pef1 is important for meiotic progression and that the activity might be altered by phosphorylation at Thr-13 and Tyr-14 during meiotic nuclear division.

### 3.2. Pef1 Regulates the Phosphorylation of Tyr-15 in Cdc2 and Promotes the Expression of DNA Replication Factors

**Figure 2:**
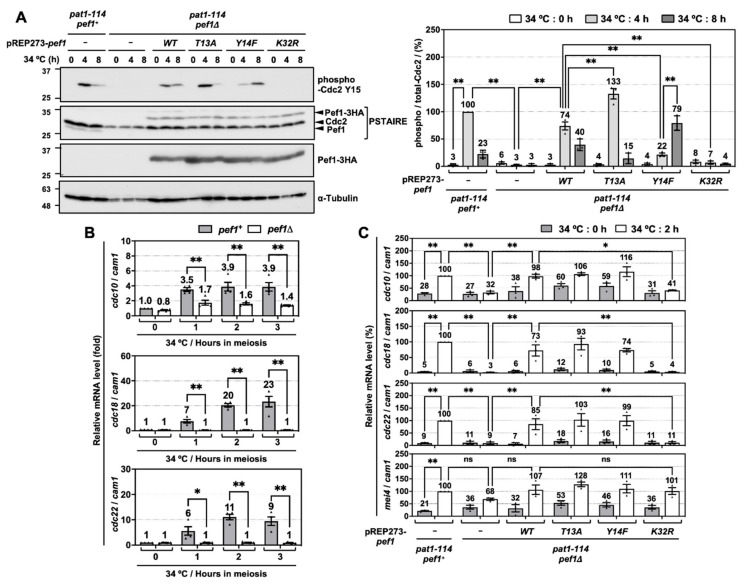
*pef1* deletion suppresses the phosphorylation of Tyr-15 in Cdc2 and expression of DNA replication factors during early-phase meiosis. (**A**) *pat1-114 pef1^+^* (FY7122) or *pat1-114 pef1Δ* (MS254) haploid cells transformed with pREP273-empty vector, pREP273-*pef1^+^*, pREP273-*pef1^T13A^*, pREP273-*pef1^Y14F^* or pREP273-*pef1^K32R^* were synchronized at the G1 phase by nitrogen starvation. Synchronous meiosis was induced by shifting the cultures from a permissive temperature (25 °C) to a restrictive temperature (34 °C). The cells were fixed at the indicated times and then subjected to western blot analysis using anti-phospho-Cdc2 Y15, anti-PSTAIRE and anti-HA antibodies. α-Tubulin was monitored as a loading control. The graph indicates the percentages of normalized phosphorylated-Cdc2 Y15. Results are expressed as means ± SEM from three independent experiments. The intensity of the normalized band in *pat1-114 pef1^+^* + pREP273-vector at the point of 4 h is taken as 100%. ** *p* < 0.01 (ANOVA). (**B**) *pat1-114 pef1^+^* (FY7052) and *pat1-114 pef1Δ* (MS030) were synchronized at the G1 phase by nitrogen starvation. Synchronous meiosis was induced by temperature shifting. Total RNA was extracted from these cells at 0 or 2 h after meiotic induction. cDNA was synthesized by reverse transcription and then relative mRNA levels of *cdc10*, *cdc18* and *cdc22* were analyzed by qPCR. *cam1* was used as a loading control. Results are expressed as means ± SEM from three independent experiments. The normalized mRNA level in *pat1-114 pef1^+^* at 0 h is taken as 1. ** *p* < 0.01, * 0.01 < *p* < 0.05 (ANOVA). (**C**) *pat1-114 pef1^+^* (FY7122) or *pat1-114 pef1Δ* (MS254) haploid cells were transformed with pREP273-empty vector, pREP273-*pef1^+^*, pREP273-*pef1^T13A^*, pREP273-*pef1^Y14F^* or pREP273-*pef1^K32R^*. The cells were collected after the induction of synchronous meiosis. Total RNA extracted from the cells was reverse-transcribed to cDNA and then qPCR analysis was performed using these cDNA. *cam1* was used as a loading control. Results are expressed as means ±SEM from three independent experiments. The normalized mRNA level in the *pat1-114 pef1^+^* + pREP273 vector after 2 h of temperature shifting is taken as 100%. ** *p* < 0.01; * 0.01 < *p* < 0.05; ns, not significant *p* > 0.05 (ANOVA).

To investigate the molecular mechanisms of how Pef1 regulates meiotic progression, we next examined the alteration of Tyr-15 phosphorylation in Cdc2 because proper phosphorylation of Tyr-15 is required for the completion of pre-meiotic DNA replication and entry into phase I meiosis [8]. As shown in Figure 2A, Western blot analysis using anti-PSTAIRE and anti-HA antibodies confirmed that endogenous Pef1 completely disappears in *pef1*-deficient cells and equal amounts of Pef1-3HA mutants are expressed. In control cells (*pat1-114 pef1^+^* + pREP273-vec), Tyr-15 phosphorylation of Cdc2 was increased after 4 h of meiotic induction and then decreased after 8 h. Thus, Cdc2 is temporally inactivated at the late pre-meiotic S phase. Unlike control cells, Cdc2 Tyr-15 phosphorylation was not detected during the induction of meiosis in *pef1*-deficient cells with empty vectors or *pef1^K32R^*. Conversely, in Pef1 WT-expressing *pef1*-deficient cells, phosphorylation of Tyr-15 in Cdc2 was 26% lower at 4 h and 17% higher at 8 h compared to control cells, indicating that Pef1 WT activity from the vectors was insufficient to promote phosphorylation. Additionally, Pef1 T13A expression efficiently increased Tyr-15 phosphorylation in Cdc2 at 4 h to the same level as in control cells. It decreased sufficiently at 8 h later, whereas phosphorylation in Pef1 Y14F-expressing cells was 78% lower at 4 h and 56% higher at 8 h compared to in control cells. These results suggest that Pef1 controls the timing of meiosis I entry by modulating the phosphorylation status of Cdc2. Furthermore, it is expected that dephosphorylation of Thr-13 and phosphorylation of Tyr-14 in Pef1 are important for the progression of meiosis.

To obtain further information on the regulatory mechanisms of meiosis by Pef1, we next focused on DNA replication factors. Perturbation of DNA replication inactivates Cdc25 phosphatase, which is responsible for the dephosphorylation of Tyr-15 in Cdc2 [24,25,26]. In *S. pombe*, G1/S transition and DNA replication require the activation of Cdc10, a core element of the MluI cell cycle box-binding factor (MBF) complex, a functional homolog of mammalian E2F [27,28]. The activated MBF complex promotes the expression of genes required to complete the S phase, such as *cdc18* and *cdc22* [29,30,31] and its activity is suppressed by cyclin Cig2-mediated phosphorylation of Res1 and co-suppressor Nrm1/Yox1 in the mitotic S phase [32,33,34,35]. To examine the activity of MBF in meiosis, we constructed specific primers for each gene and performed reverse-transcriptional quantitative real-time PCR (RT-qPCR). RT-qPCR analysis demonstrated that *cdc10* expression levels and DNA replication factors (*cdc18* and *cdc22*) increased rapidly in response to meiotic induction. In contrast, these genes were silenced at all times in *pef1*-deficient cells (Figure 2B). We also examined the effect of introducing Pef1 mutants on the expression of these genes and found that *cdc10*, *cdc18* and *cdc22* expression levels significantly increased after 2 h of meiotic induction in control cells (Figure 2C). In contrast, the expression of these genes was silenced in *pef1*-deleted cells and Pef1 K32R-expressing cells. Interestingly, these genes silencing were significantly recovered in Pef1 WT-, T13A- and Y14F-expressing cells. However, there were no differences in the gene expression levels among Pef1 WT and mutants, suggesting that phosphorylation of Pef1 is unaffected by the expression of these genes in the initiation of meiosis. We also tested whether *mei4*, which encodes a forkhead transcription factor, is regulated by *pef1*, because Mei4 promotes the expression of various meiotic genes, such as *cdc25*, encoding a phosphatase that dephosphorylates Tyr-15 of Cdc2 [36,37]. Surprisingly, *pef1* deletion slightly decreased the expression level of *mei4* mRNA at the early phase of meiotic induction (2 h after temperature shifting) compared with that of control cells. However, there were no significant differences in the expression level between those cells and each of the Pef1 mutant-(WT, T13A, Y14F and K32R) expressing cells (Figure 2C). These results indicate that Pef1 promotes the expression of DNA replication factors, resulting in the increased phosphorylation of Tyr-15 in Cdc2.

Our data also indicate that the phosphorylated form of Pef1 modulates the efficiency of Cdc2 phosphorylation of Tyr-15 through a DNA replication-independent pathway. That is, it is expected that Pef1 directly regulates the activity of kinases or phosphatases such as Cdc25 or Wee1. From these results, we can posit that Pef1 contributes to meiosis I entry via a dual cascade: promoting the expression and activation of Cdc10 and controlling the phosphorylation status of Tyr-15 in Cdc2.

### 3.3. Pef1 Deletion Interrupts Pre-Meiotic Chromosomal DNA Replication 

**Figure 3:**
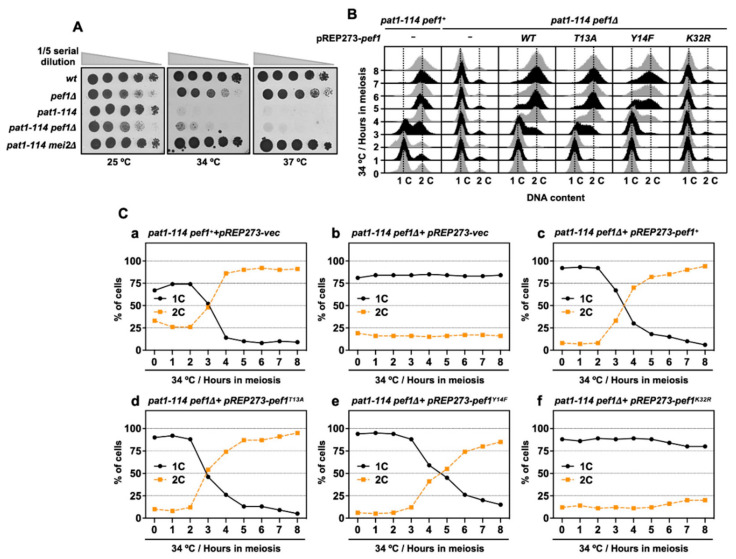
*pef1* deletion blocks pre-meiotic DNA replication. (**A**) *wt* (L972), *pef1Δ* (MS009), *pat1-114* (FY7052), *pat1-114 pef1Δ* (MS030) and *pat1-114 mei2Δ* (AN0579) haploid cells were precultured in EMM liquid medium. Cultures serially diluted five times were spotted on the EMM agar medium and incubated at the indicated temperatures for 3–5 days. (**B**,**C**) *pat1-114 pef1^+^* (AN0353) or *pat1-114 pef1Δ* (MS254) cells were transformed with pREP273-empty vector (Ca, Cb), pREP273-*pef1^+^* (Cc), pREP273-*pef1^T13A^* (Cd), pREP273-*pef1^Y14F^* (Ce)or pREP273-*pef1^K32R^*(Cf). Synchronous meiosis was induced by temperature shifting. The cells were fixed at the indicated times and stained with propidium iodate (PI). DNA contents were measured by flow cytometry analysis (**B**). Percentages of cells with an unreplicated DNA content (1C, black solid line) and replicated DNA content (2C, orange broken line) after the induction of synchronous meiosis. Results are expressed as averages from two independent experiments (**C**).

Inactivation of Pat1 kinase leads to the accumulation of Mei2, which is essential for meiotic initiation [6]. Thus, the *pat1-114 mei2Δ* strain can grow even in high temperatures (34 °C and 37 °C) because this strain cannot initiate meiosis. *pef1* deletion does not affect the *pat1-114* temperature sensitivity of growth (Figure 3A) and it is considered that meiosis is arrested before entry into meiosis I in *pat1-114 pef1Δ* cells. Furthermore, our RT-qPCR analysis showed that DNA replication factors were silenced by *pef1* deletion (Figure 2C); thus, we suspected that *pef1* controls the pre-meiotic replication of chromosomal DNA. To examine whether chromosomal DNA is replicated in *pef1*-deleted cells, we measured the DNA content by flow cytometry analysis. As shown in Figs. 3B and C, the DNA content was increased 3 h after induction of synchronous meiosis in control cells (Figure 3(Ca)); however, in cells that had lost the *pef1* gene or Pef1 activity, the DNA content was not increased after meiotic induction (Figure 3(Cb,f)). Corresponding to the results shown in Figure 1(Cc), the expression of Pef1 WT provided the driving force for the progression of meiosis in *pef1-*deleted cells (Figure 3(Cc)) and this driving force was activated by the expression of Pef1 T13A (Figure 3(Cd)) and inactivated by the expression of Pef1 Y14F (Figure 3(Ce)). Together, these results indicate that *pef1* regulates DNA replication in the early pre-meiotic S phase. Moreover, the efficiency of completing DNA replication depends on the phosphorylation status of Pef1 and the status of meiotic nuclear division (Figure 1C) and Tyr-15 phosphorylation (Figure 2A).

### 3.4. Double Deletion of clg1 and psl1 Disturbs Meiotic Nuclear Division

**Figure 4:**
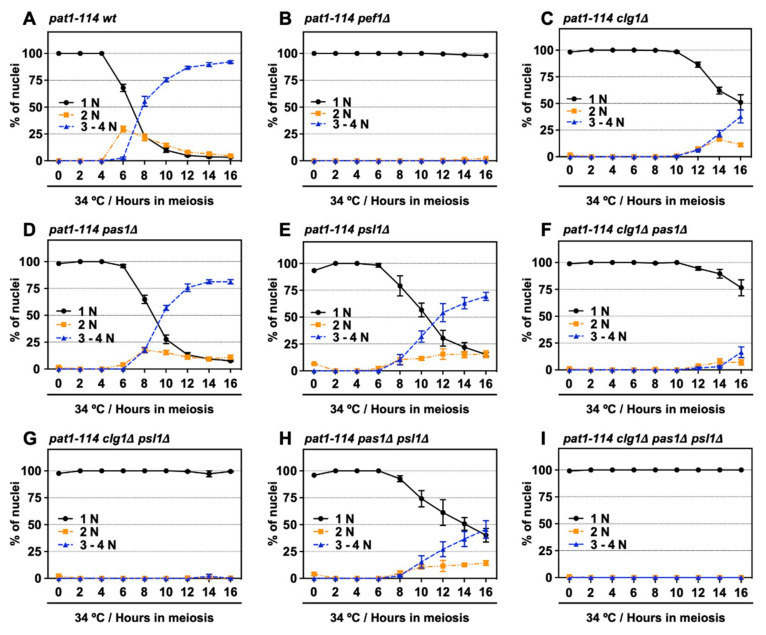
Double deletion of *clg1* and *psl1* strongly suppresses meiotic progression. (**A**–**I**) *pat1-114 wt* (FY7052) (**A**), *pat1-114 pef1Δ* (MS030) (**B**), *pat1-114 clg1Δ* (MS105) (**C**), *pat1-114 pas1Δ* (MS106) (**D**), *pat1-114 psl1Δ* (MS107) (**E**), *pat1-114 clg1Δ pas1Δ* (MS155) (**F**), *pat1-114 clg1Δ psl1Δ* (MS119-1) (**G**), *pat1-114 pas1Δ psl1Δ* (MS156) (**H**) and *pat1-114 clg1Δ pas1Δ psl1Δ* (MS126-2) (**I**) were nitrogen-starved for 14–16 h. Synchronous meiosis was induced by shifting the cultures from a permissive temperature (25 °C) to a restrictive temperature (34 °C). The cells were fixed at the indicated times and then stained with DAPI. The graph indicates percentages of the G1-pre-meiotic S phase (1N, black solid line), meiosis (2N, orange broken line) and meiosis and ascus (3–4N, blue broken line). At least 100 cells were counted each time. The results are expressed as means ± SEM from three independent experiments.

We have previously shown that Pef1 interacts with three cyclins: Clg1, Pas1 and Psl1 [18]. Our genetic analysis also indicated that Clg1 and Pas1 act as activators of Pef1 during vegetative growth but little information regarding these cyclins’ function in meiosis has been available. Thus, we verified whether these cyclins activate Pef1 in meiosis. First, we constructed a series of cyclin-deficient *pat1-114* strains and evaluated the effects of cyclin deletion on meiotic progression efficiency. The temperature sensitivity of these cyclin-deficient *pat1-114* strains was confirmed by spot assay on EMM agar (Figure S2A,B). As shown in Figure 4A and Figure S2C, the control cells (*pat1-114 wt*) efficiently entered into meiosis I and meiosis II after 6 h of meiotic induction, whereas meiotic cells were not observed in *pef1*-deficient cells (Figure 4B and Figure S2C). Interestingly, meiosis was drastically delayed in *clg1*-deficient cells (3–4N cells were observed after 12 h of meiotic induction) compared with in control cells (Figure 4C and Figure S2C). Meiotic initiation in *pas1-* and *psl1-*deficient cells was slightly delayed compared to the controls (Figure 4D,E and Figure S2C; 3–4N cells were observed after 8 h of meiotic induction) and double gene deletion of *clg1Δ pas1Δ*, *clg1Δ psl1Δ and pas1Δ psl1Δ* synergistically delayed meiotic initiation (Figure 4F–H and Figure S2D), suggesting that these cyclins contribute to meiotic initiation and progression. Moreover, because *clg1Δ psl1Δ* cells, as well as *clg1Δ* cells, showed no ability to initiate meiosis during at least 16 h of induction (Figure 4G and Figure S2D), Clg1 and Psl1 are considered to be activators of Pef1 during meiosis.

### 3.5. Cdc2 Phosphorylation and DNA Replication Factors Are Silenced by the Double Deletion of clg1 and psl1

**Figure 5:**
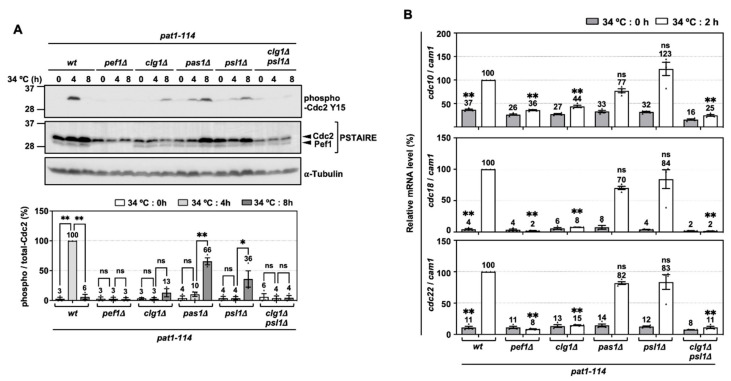
Phosphorylation of Tyr-15 in Cdc2 and expression of DNA replication factors are strongly silenced by deletion of *clg1* and *psl1*. (**A**) Synchronous meiosis of *pat1-114 wt* (FY7052), *pat1-114 pef1Δ* (MS030), *pat1-114 clg1Δ* (MS105), *pat1-114 pas1Δ* (MS106), *pat1-114 psl1Δ* (MS107) and *pat1-114 clg1Δ psl1Δ* (MS119-1) was induced by nitrogen starvation, followed by temperature shifting from 25 °C to 34 °C. The cells were fixed at the indicated times and then subjected to western blot analysis using anti-phospho-Cdc2 Y15 and anti-PSTAIRE antibodies. α-Tubulin was monitored as a loading control. The graph indicates the percentages of normalized phosphorylated Cdc2 Y15. The results are expressed as means ±SEM from three independent experiments. The intensity of the normalized band in *pat1-114 pef1^+^* + pREP273-vector at 4 h is taken as 100%. ** *p* < 0.01; * 0.01 < *p* < 0.05; ns, not significant *p* > 0.05 (ANOVA). (**B**) Total RNA extracted from meiosis-induced cells reverse-transcribed to cDNA and subjected to RT-qPCR analysis. *cam1* was used as a loading control. The results are expressed as means ±SEM from three independent experiments. The normalized mRNA level in *pat1-114 pef1^+^* + pREP273 vector at 2 h is taken as 100%. ** *p* < 0.01; * 0.01 < *p* < 0.05; ns, not significant *p* > 0.05 (ANOVA).

To verify that *clg1* and *psl1* are activators of *pef1* in meiosis, we examined whether these cyclins are involved in regulating Cdc2 phosphorylation and the expression of DNA replication factors in the pre-meiotic S phase. As shown in Figure 5A, the phosphorylation of Tyr-15 in Cdc2 peaked after 4 h of meiotic induction in control cells. It was significantly suppressed to the same extent as *pef1Δ* cells by the double deletion of *clg1* and *psl1*. The peak phosphorylation of Tyr-15 in Cdc2 was also delayed after the single deletion of *clg1*, *pas1 and psl1* (peaked at 8 h of meiotic induction), suggesting that Clg1, Pas1 and Psl1 contribute to the regulation of Cdc2 phosphorylation of Tyr-15. RT-qPCR analysis also showed that the expression levels of *cdc10*, *cdc18* and *cdc22* during meiosis were strongly silenced in *clg1Δ psl1Δ* cells compared to in the control cells (Figure 5B). In addition, these genes were also significantly suppressed by the single deletion of *clg1* but not the single deletions of *pas1* or *psl1.* These results indicate that Clg1 and Psl1 are activators of Pef1 in meiosis because the phenotypes of *clg1Δ psl1Δ* cells are similar to those of *pef1Δ* cells. Furthermore, our data imply that Pas1 and Psl1 regulate the phosphorylation of Tyr-15 in Cdc2 during the pre-meiotic S phase, whereas Clg1 controls the upregulation of both the phosphorylation of Cdc2 and DNA replication factors.

### 3.6. Deletion of clg1 Stalls Initiation of Pre-Meiotic DNA Replication

**Figure 6:**
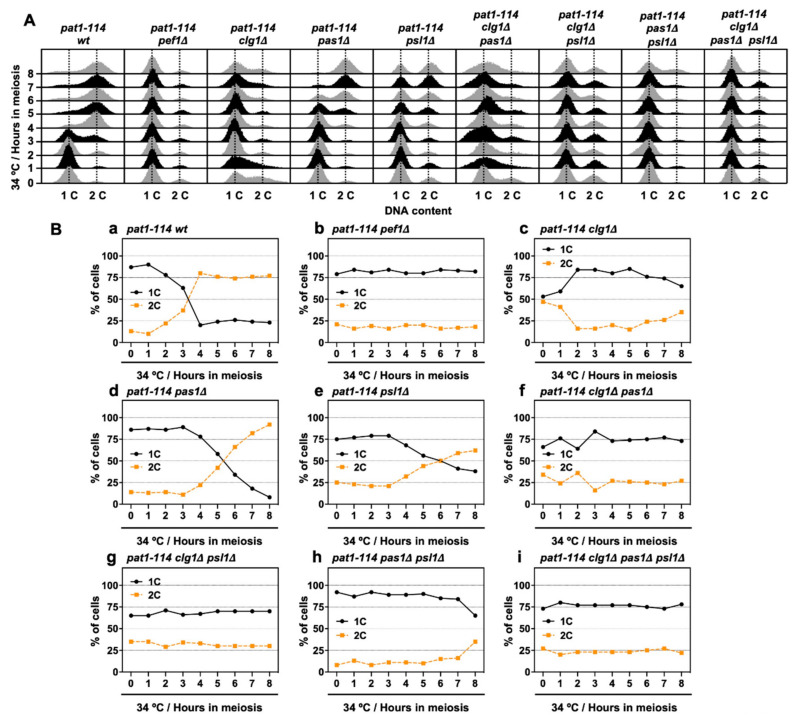
*clg1* deletion blocks pre-meiotic DNA replication. (**A**,**B**) *pat1-114 wt* (FY7052) (Ba), *pat1-114 pef1Δ* (MS030) (Bb), *pat1-114 clg1Δ* (MS105) (Bc), *pat1-114 pas1Δ* (MS106) (Bd), *pat1-114 psl1Δ* (MS107) (Be), *pat1-114 clg1Δ pas1Δ* (MS155) (Bf), *pat1-114 clg1Δ psl1Δ* (MS119-1) (Bg), *pat1-114 pas1Δ psl1Δ* (MS156) (Bh) and *pat1-114 clg1Δ pas1Δ psl1Δ* (MS126-2) (Bi) were nitrogen-starved for 14–16 h. Synchronous meiosis was induced by shifting cultures from a permissive temperature (25 °C) to a restrictive temperature (34 °C). The cells were fixed at the indicated times and then stained with PI. DNA contents were measured by flow cytometry analysis (**A**). Graphs indicate the percentages of cells with an unreplicated DNA content (1C, black solid line) and replicated DNA content (2C, orange broken line) after the induction of synchronous meiosis (**B**). Results are expressed as averages from two independent experiments.

Next, we performed flow cytometry analysis to determine whether chromosomal DNA is replicated during meiosis in cyclin-deficient cells. Figure 6A shows the histograms from the flow cytometry analysis. The flow cytometry analysis revealed that nitrogen starvation effectively leads more than 75% of cells to the G1 phase (Figure 6(Ba,b,d,e,h)), except for *clg1Δ* cells (1C: 53%) (Figure 6(Bc)), implying that *clg1* deletion adversely affects nitrogen starvation-induced G1 arrest. However, the insufficient G1 arrest was restored in *clg1Δ pas1Δ* (1C: 66%), *clg1Δ psl1Δ* (1C: 65%) and *clg1Δ pas1Δ psl1Δ* cells (1C: 73%) (Figure 6(Bf,g,i)) when compared with that of *clg1Δ* cells. Also, the percentage of duplicated DNA (2C) exceeded 50% after 4–6 h of meiotic induction in control, *pas1Δ* and *psl1Δ* cells (Figure 6(Ba,d,e)) but was not altered in *clg1Δ* cells during the time course of meiotic induction (Figure 6(Bc)). Although double (*clg1Δ pas1Δ* and *clg1Δ psl1Δ*) and triple (*clg1Δ pas1Δ psl1Δ*) mutants were also unable to increase the DNA content after meiotic induction (Figure 6(Bf,g,i)), the percentage of 2C in *pas1Δ psl1Δ* double mutants increased after 8 h of meiotic induction (Figure 6(Bh)). The flow cytometry analysis results revealed that Clg1 is required for G1 arrest and the initiation of DNA replication in the early pre-meiotic S phase. Pas1 and Psl1 regulate DNA replication efficiency and play a potential opposite role to Clg1 in the regulation of G1 arrest. 

## 4. Discussion

**Figure 7:**
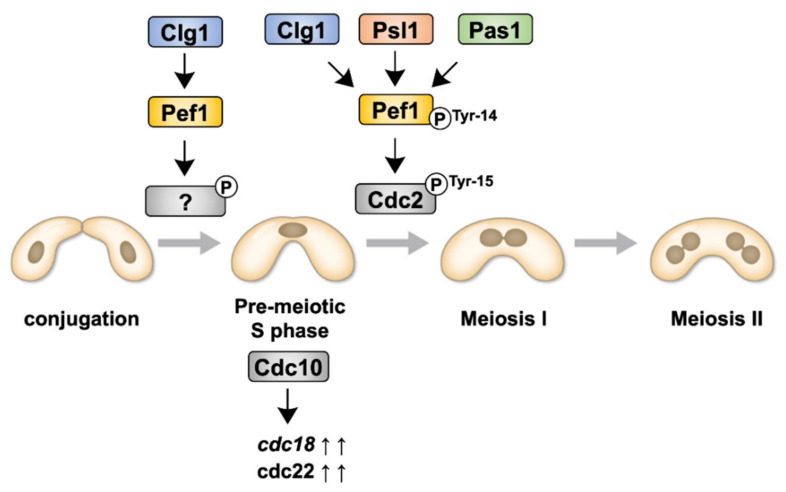
Illustration representing the signaling pathways of Pef1 and cyclins during meiosis.

Although we have previously reported that Pef1 regulates sexual differentiation by regulating the TORC1, autophagy and Ste11–Mei2 pathways [18], the physiological roles of Pef1 during meiosis are, as yet, unresolved. In this study, we have demonstrated that Pef1 is essential for meiotic progression. Figure 7 presents the signaling pathway of Pef1 in meiosis to allow easier interpretation of the results. Pef1 and Clg1 are required for the upregulation of DNA replication factors. Clg1, Psl1 and Pas1 also contribute to meiotic I entry by promoting the Cdc2 phosphorylation at Tyr-15. The phosphorylation of Pef1 at Tyr-14 is also considered required to accelerate Cdc2 phosphorylation at Tyr-15.

Pef1 is an orthologue of the vertebrate CDK5 protein. Intriguingly, mouse CDK5 interacts with its activator, p35 and exhibits kinase activity in the ovaries; therefore, the CDK5/p35 complex is considered involved in regulating ovarian function [38]. However, little is known about the precise function of CDK5 in germline cells, even though mammalian CDK5 is ubiquitously expressed in various tissues, including the testes and ovaries [39]. CDK16, a CDK family member that is very similar to CDK5, is required for spermatogenesis [40] but its underlying mechanism has not yet been determined. Thus, our study provides novel insights into the regulatory mechanisms of meiosis and germ cell development by CDK5 and its closely related kinases. 

In the fission yeast *S. pombe*, the initiation programs of meiosis are actuated by the accumulation of Mei2, which is triggered by nutrient deprivation. Periodic oscillation of Cdc2 activity is required to facilitate and complete a meiotic division [8]. In meiosis, Cdc2 activity is modulated through interaction with cyclins, such as Cig2, Rem1 and Cdc13 and modification of Tyr-15 phosphorylation status by Wee1 kinase and Cdc25 phosphatase [8,10,41,42]. Mei4 induces the expression of Wee1 and Cdc25, a forkhead transcription factor that regulates gene expression during the middle phase of meiosis [36,37,43]. Our analysis demonstrates that Pef1 is unrelated to the regulation of Mei4 expression, whereas the phosphorylation of Tyr-15 in Cdc2 is modulated by Pef1 (Figure 2A). This finding indicates that Pef1 controls the phosphorylation of Tyr-15 in Cdc2 by regulating Wee1 and Cdc25 directly or indirectly. Intriguingly, mouse CDK5/p25 directly phosphorylates the Cdc25 isoform Cdc25A-C in vitro and phosphorylation causes hyperactivation of phosphatase activity, resulting in neural death by CDK activation [44]. Thus, Pef1 seems to directly modulate Cdc25 phosphatase activity, such as that of mouse CDK5. Conversely, in *S. pombe*, the TORC1-Greatwall-PP2A pathway also controls the phosphorylation status of Cdc2 Tyr-15 by activating Wee1 and inactivating Cdc25 [45]. In addition to TORC1, Pho85, an orthologue of Pef1 in *S. cerevisiae*, suppresses Greatwall kinase Rim15 to regulate autophagy [46]. Because we have recently shown that Pef1 positively regulates TORC1 activity [18], it is expected that Pef1 modulates Cdc2 Tyr-15 phosphorylation through the TORC1-Greatwall-PP2A pathway during meiosis.

Pef1 contains predicted phosphorylation sites at Thr-13 and Tyr-14 in its amino acid sequence, which correspond to Thr-14 and Tyr-15 in Cdc2. Phosphorylation of Pef1 is not required to initiate pre-meiotic DNA replication because the expression of Pef1 T13A and Y14F does not affect the expression of DNA replication factors (Figure 3B). However, our analyses on the effects of meiotic progression using *pat1-114* mutants show that expression of the hyperactive version of Pef1 (Pef1 T13A) accelerates Cdc2 Tyr-15 phosphorylation compared to that of the Pef1 wild-type. In contrast, the less active version of Pef1 (Pef1 Y14F) delays the timing of Cdc2 Tyr-15 phosphorylation during synchronous meiosis (Figure 2A). These results suggest that the phosphorylation of these sites also regulates Pef1 activity; that is, Pef1 activity is decreased by Thr-13 phosphorylation and increased by Tyr-14 phosphorylation in the progression of the pre-meiotic S phase. Notably, some studies have indicated that mammalian CDK5 is phosphorylated at Tyr-15 by the Src family or receptor-type tyrosine kinases [47,48,49,50,51]. However, after a recent work demonstrated that phosphorylation of Tyr-15 in CDK5 competes with its activators p35, p25 and cyclin I [52], it is considered that phosphorylation is not required for CDK5 activation in mammals. Therefore, the mechanisms and physiological significance of Pef1 phosphorylation should be carefully considered, as well as that of mammalian CDK5.

We also found that Pef1 and Clg1 promote pre-meiotic DNA replication. In early-phase meiosis, it has been predicted that Cdc10 triggers the expression of genes required for DNA replication and recombination by associating with Rep1 (Rec16), a meiotic-specific MBF activator [29,53]. In addition, Rep1 has been identified as a target of Ste11, a master transcriptional regulator of early sexual differentiation [54]. Because we have previously shown that Pef1 is required to upregulate *ste11* during nitrogen starvation [18], we considered that the silencing of *cdc18* and *cdc22* in *pef1*-deficient cells during the pre-meiotic S phase was due to not only the suppression of *cdc10* expression but also the lack of Ste11-mediated Rep1 expression. Thus, it is predicted that Pef1 regulates both the expression and the MBF complex activity during meiosis.

Furthermore, nuclear importation of Ste11 is important for gene expression and is suppressed by TORC1 under nutrient-rich conditions [55]. Although nitrogen starvation generally promotes nuclear accumulation of Ste11, the Ste11 content localized in the nucleus decreased in *pef1*-deficient cells during nitrogen starvation [18]. We found that these irregular Ste11 responses were caused by excessive TORC1 reactivation during nitrogen starvation. Thus, it is considered that misregulation of the TORC1-Ste11-Mei2 pathway caused by loss of *pef1* is responsible for the silencing of *cdc10* and DNA replication factors *cdc18* and *cdc22* in the pre-meiotic S phase.

This study also provides new insights into the functions of cyclins. In mammals, CDK5 phosphorylates its activator, p35, to suppress the conversion of p35 to p25 by calpain-dependent cleavage. Because p25 induces irregular activation of CDK5, which causes neural disease, precise regulation of p35 phosphorylation is important for maintaining the homeostasis of neural function [56]; thus, it is considered that the stability of Clg1, Psl1 and Pas1 are also controlled by Pef1 or other kinases. Remarkably, Clg1 retains the destruction box (D-box) motif (R*xx*L*xxxx*N/D/E, where *x* represents any residue) in its amino acid sequence (residues 365–373, RTALLNSFE). Because the D-box is a possible recognition motif of anaphase-promoting complex/cyclosome (APC/C) [57,58], Clg1 may be degraded by the ubiquitin-proteasome system. Conversely, Psl1 and Pas1 do not have D-boxes in their amino acid sequences; thus, it is considered that the ubiquitin-proteasome system does not target Psl1 and Pas1. Therefore, it is intriguing to examine the stability and ubiquitination of these cyclins in meiosis.

## 5. Conclusions

Meiosis is a specialized cell division process that mediates the transfer of genetic information to the next generation. We found that Pef1 promotes DNA replication and phosphorylation of Tyr-15 in Cdc2 during meiosis. Three cyclins: Clg1, Psl1 and Pas1 also contribute to the proper progression of meiosis. This study provides valuable information about the regulatory mechanisms of the initiation and progression of meiosis in *S. pombe*.

## Figures and Tables

**Table 1:** *S. pombe* strains used in this study.

Strain	Genotype	Source/Reference
L972	*h^−^*	National-Bio-Resource-Project
FY7052	*h^−^ pat1-114*	National-Bio-Resource-Project
FY7122	*h^−^ pat1-114 leu1^-^*	National-Bio-Resource-Project
AN0353	*h^−^/h^+^ ade6-M210/ade6-M216*	This study
AN0579	*h^−^ pat1-114 mei2::hphMX*	This study
MS009	*h^-^ pef1::hphMX*	[18]
MS030	*h^−^ pat1-114 pef1::hphMX*	This study
MS053	*h^−^ pat1-114 pef1-3HA:hphMX*	This study
MS065	*h^−^ psl1::hphMX*	[18]
MS067	*h^−^ clg1::hphMX*	[18]
MS069	*h^−^ pas1::hphMX*	[18]
MS073	*h^+^ pef1::natR ade6M210*	This study
MS100	*h^−^/h^+^ pef1::hphMX/pef1::natR ade6M210 diploid*	This study
MS113	*h^-^ clg1::hphMX psl1::hphMX*	[18]
MS115	*h^−^ clg1::hphMX pas1::kanMX psl1::hphMX*	[18]
MS157	*h^−^ pas1::KanMX psl1::hphMX*	[18]
MS159	*h^−^ pas1::KanMX clg1::hphMX*	[18]
MS105	*h^−^ pat1-114 clg1::hphMX*	This study
MS106	*h^−^ pat1-114 pas1::kanMX*	This study
MS107	*h^−^ pat1-114 psl1::hphMX*	This study
MS119-1	*h^−^ pat1-114 clg1::hphMX psl1::hphMX*	This study
MS126-2	*h^−^ pat1-114 clg1::hphMX pas1::kanMX psl1::hphMX*	This study
MS155	*h^−^ pat1-114 clg1::hphMX pas1::kanMX*	This study
MS156	*h^−^ pat1-114 psl1::kanMX pas1::kanMX*	This study
MS254	*h^−^ pat1-114 leu1^-^ pef1::hphMX*	This study

**Table 2:** List of primers used for Reverse-transcription Quantitative Real-Time PCR (RT-qPCR).

Gene	Forward Primer (5′ to 3′)	Reverse Primer (5′ to 3′)
*cdc10*	AACAAACTTTGGCAACGAAA	AACACGTAAGAATCCAGCAA
*cdc18*	TTTGTGTCTTCGAGATCGTT	TGTCTCCAACAGCTGTAATG
*cdc22*	TTTTATGGCTGGAAGAAGGG	CTCTTCTTTAGTGGGCTCTG
*mei4*	ATGTCCCTGATTTAACACCC	TGATTAAGTTCAGGGCTGTC
*cam1*	CCCGAAAAATGAAGGATACC	CTTGGAAGAAATGACACGAG

## Data Availability

There is no data availability statement.

## Supplementary Materials

The following are available online at https://www.mdpi.com/2218-273X/11/1/89/s1, Figure S1: Meiosis and sporulation in *pef1*-deleted cells, Figure S2: Temperature sensitivity of cyclin-deleted *pat1-114* cells.
