# Supplementary material for: The S. pombe CDK5 Orthologue Pef1 Cooperates with Three Cyclins, Clg1, Pas1 and Psl1, to Promote Pre-Meiotic DNA Replication
Source: Biomolecules. 2021 Jan 12;11(1):89. doi: 10.3390/biom11010089 (PMC7828282; doi:10.3390/biom11010089)
Supplement: Supplementary file 1 [file biomolecules-11-00089-s001.pdf]

Supplemental Figure

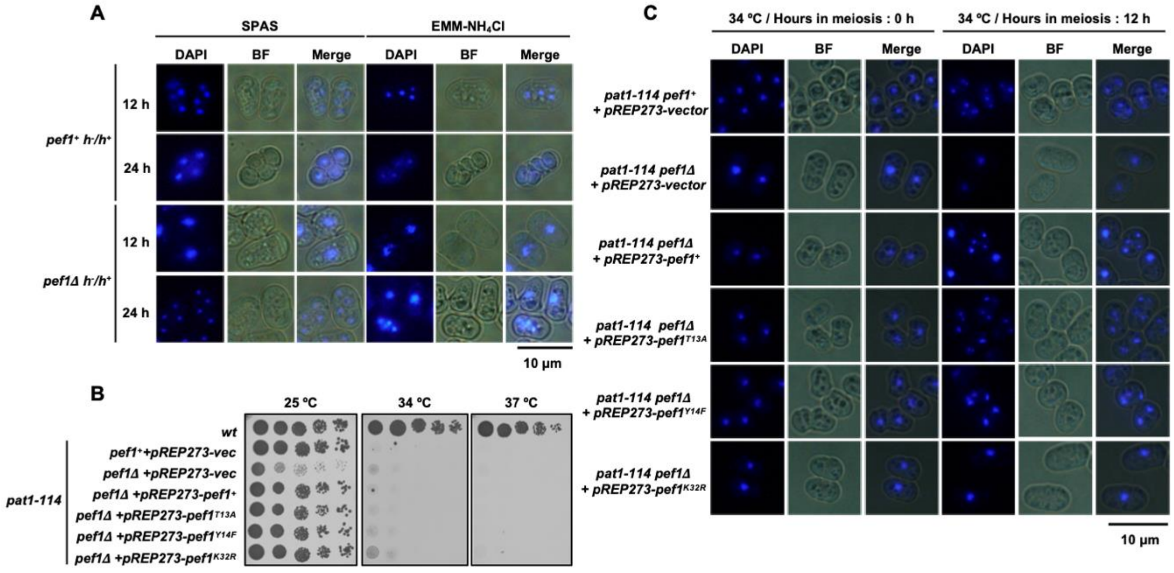

Figure S1. Meiosis and sporulation in *pef1*-deleted cells. (A) *wt* (AN0353) and *pef1Δ* (MS100) diploid cells grown in YES at 33°C were incubated on SPAS or EMM–NH_4Cl agar for the indicated times at 25°C. Cells were fixed, stained with DAPI, and observed using fluorescence microscopy. Fluorescence images of DAPI are shown as blue. BF: Bright-field. Scale bars: 10 μm. (B, C) *pat1-114 pef1*$^{+}$ (FY7122) or *pat1-114 pef1Δ* (MS254) haploid cells transformed with pREP273-empty vector, pREP273-*pef1*$^{+}$, pREP273-*pef1*^{T13A}, pREP273-*pef1*^{Y14F}, or pREP273-*pef1*^{K32R}. Cultures serially diluted five times (*wt*: L972) were spotted on EMM agar and incubated at 25°C, 34°C, or 37°C (B). Synchronous meiosis was induced by nitrogen starvation, followed by a temperature shift from permissive (25°C) to restrictive (34°C). The cells were fixed at the indicated times, stained with DAPI, and observed using fluorescence microscopy. Fluorescence images of DAPI are shown as blue. BF: Bright-field. Scale bars: 10 μm (C).

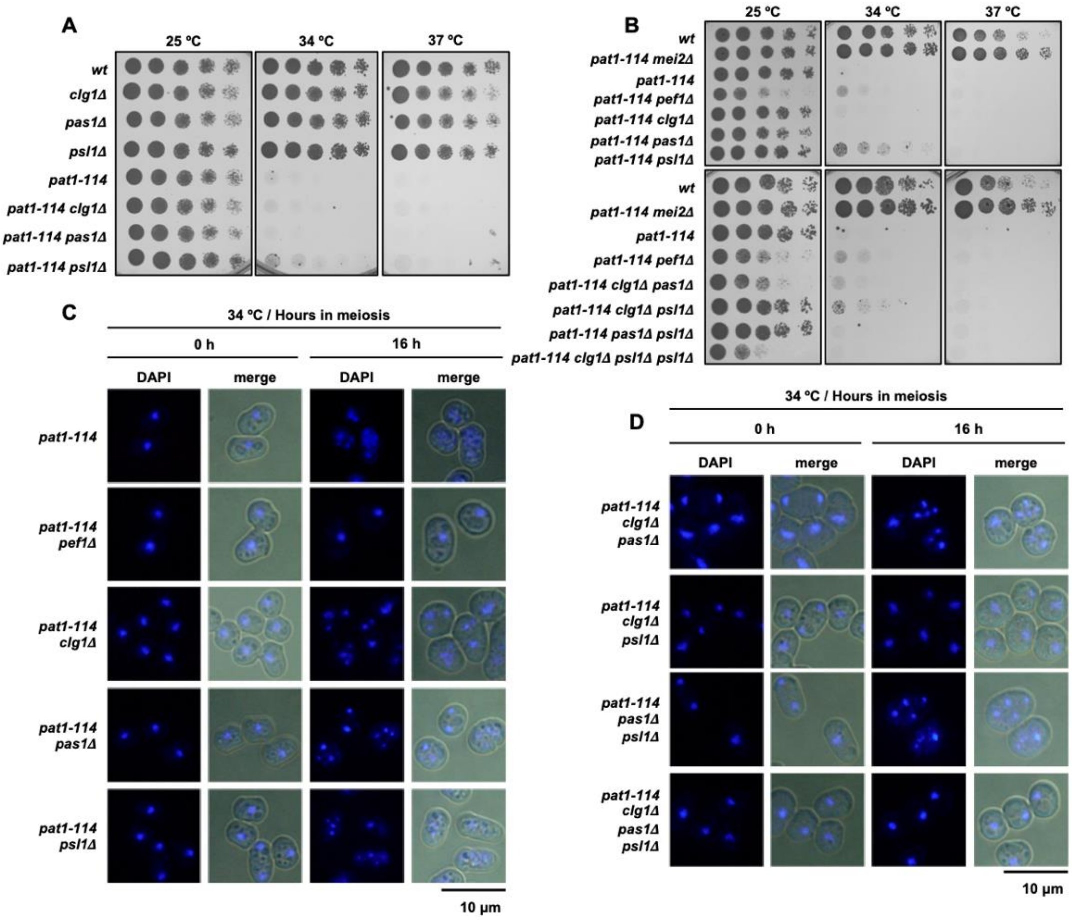

Figure S2. Temperature sensitivity of cyclin-deleted *pat1-114* cells. (A, B) *wt* (L972), *pat1-114 mei2Δ* (AN0579), *clg1Δ* (MS067), *pas1Δ* (MS069), *psl1Δ* (MS065), *pat1-114* (FY7052), *pat1-114 pef1Δ* (MS030), *pat1-114 clg1Δ* (MS105), *pat1-114 pas1Δ* (MS106), *pat1-114 psl1Δ* (MS107), *pat1-114 clg1Δ pas1Δ* (MS155), *pat1-114 clg1Δ psl1Δ* (MS119-1), *pat1-114 pas1Δ psl1Δ* (MS156), and *pat1-114 clg1Δ pas1Δ psl1Δ* (MS126-2) cells were grown on the EMM agar at the indicated temperatures. (C, D) *pat1-114 wt* (FY7052), *pat1-114 pef1Δ* (MS030), *pat1-114 clg1Δ* (MS105), *pat1-114 pas1Δ* (MS106), *pat1-114 psl1Δ* (MS107), *pat1-114 clg1Δ pas1Δ* (MS155), *pat1-114 clg1Δ psl1Δ* (MS119-1), *pat1-114 pas1Δ psl1Δ* (MS156), and *pat1-114 clg1Δ pas1Δ psl1Δ* (MS126-2) were nitrogen-starved for 16 hours. Synchronous meiosis was induced by shifting cultures from a permissive temperature (25°C) to a restrictive temperature (34°C). The cells were fixed at the indicated times and then stained with DAPI. The images were obtained using fluorescence microscopy. DAPI-stained nuclei are shown as blue. BF: Bright-field. Scale bar: 10 μm.
